# Posture-related ocular cyclotorsion during cataract surgery with an ocular registration system

**DOI:** 10.1038/s41598-020-59118-9

**Published:** 2020-02-07

**Authors:** Ryo Terauchi, Hiroshi Horiguchi, Tomoichiro Ogawa, Takuya Shiba, Hiroshi Tsuneoka, Tadashi Nakano

**Affiliations:** 1 Department of Ophthalmology, The Jikei University School of Medicine, Tokyo, Japan; 2 Miyamaedaira Ogawa Eye Clinic, Kanagawa, Japan; 3 Roppongi Shiba Eye Clinic, Tokyo, Japan

**Keywords:** Lens diseases, Refractive errors, Clinical trials

## Abstract

Ocular cyclotorsion when a patient changes from seated to supine position in cataract surgery and factors predicting the amount of cyclotorsion were investigated using VERION system. Variables analyzed were age, gender, preoperative visual acuity, axial length, laterality of eyes, operative duration, and the direction and degree of cyclotorsion. The mean cyclotorsion of 107 eyes of 93 cataract patients was 0.98 ± 4.85 degrees (median, 1 degree; range, -11 to 12 degrees), and the median absolute value was 4 degrees (mean, 4.05 ± 2.82 degrees; range, 0 to 12 degrees). Cyclotorsion was ≥3 degrees in 68 (63.6%) eyes. Excyclotorsion occurred more frequently than incyclotorsion (50.5% vs. 43.0%). There was no cyclotorsion in seven (6.5%). Multiple regression analysis showed that gender was a significant predictive factor for the absolute value of cyclotorsion (β = 1.06, *P* = 0.041); however, the other variables had no effect on cyclotorsion. The absolute value of cyclotorsion was significantly larger in female than in male patients [median, 4 degrees and 3 degrees, respectively; mean, 4.66 ± 3.02 degrees and 3.44 ± 2.52 degrees, respectively (*P* = 0.039)]. In conclusion, cataract patients had significant posture-related ocular cyclotorsion. The amount of cyclotorsion was larger for female than male patients.

## Introduction

Rotational movement of the eye occurs when a person changes from the seated to supine position^1–5^. Preoperative examinations are conducted with the patient seated, whereas refractive procedures such as toric intraocular lens (IOL) implantation and corneal refractive surgery are conducted with the patient supine. Cyclotorsion due to postural change is called “posture-related ocular cyclotorsion,” which is a main cause of intraoperative cyclotorsion and an important factor in the correction of astigmatism in refractive procedures^6–8^. Axial misalignment of 10 degrees in refractive surgery theoretically causes a 30% or greater loss in astigmatic correction^3,9^.

Previous studies measured posture-related cyclotorsion using various techniques such as Jackson cross cylinder^1^, Maddox double rod measurements^2^, videokeratography^10^, handheld keratometer^11^, and three-dimensional infrared video-oculography^4^. An iris registration system has recently been developed to track, calculate, and compensate any patient’s eye movements, including cyclotorsion, in real time. This registration system allows a more accurate measurement of the amount of cyclotorsion, compared with some previous methods^6,12,13^.

Toric IOL reduces spherical errors and astigmatic errors after cataract surgery. Reducing residual astigmatism after cataract surgery improves patient satisfaction, including independence from wearing glasses. Posture-related ocular cyclotorsion is a main cause of misalignment of a toric IOL. Therefore, accurately investigating posture-related cyclotorsion and exploring the predictive factors among cataract patients are clinically essential. Many studies have shown the degree and direction of cyclotorsion by using an ocular registration system^14–25^; however, nearly all of these studies have targeted only normal individuals or patients undergoing corneal refractive surgeries such as laser *in situ* keratomileusis (LASIK) and photorefractive keratectomy (PRK). The background (e.g., age and preoperative visual acuity) of patients undergoing excimer laser refractive surgery is completely different from that of cataract patients; therefore, the elucidation of posture-related cyclotorsion of patients with cataract is desired. Suzuki *et al*.^10^ demonstrated posture-related cyclotorsion in 16 cataract patients using videokeratography. Hummel *et al*.^24^ reported cyclotorsion in cataract patients by using an ocular registration system. They measured cyclotorsion during femtosecond laser-assisted cataract surgery, which affects measured values by the docking process with the suction ring (i.e., patient interface). To the best of our knowledge, accurately measuring posture-related cyclotorsion in patients undergoing conventional cataract surgery by using an ocular registration system has not been reported.

In this paper, we measured posture-related cyclotorsion in phacoemulsification surgery by using the VERION Image Guided System (Alcon Laboratories, Ft. Worth, TX, USA). Lin *et al*.^26^ indicate that the VERION system is a useful tool for accurately measuring rotational ocular movement in cataract patients undergoing toric IOL implantation. This system can calculate the direction and degree of cyclotorsion by synchronizing the image of the eyes of a patient while supine with the anterior ocular segment image that was captured preoperatively with the patient seated. Furthermore, we explored the factors affecting the absolute value of cyclotorsion.

## Results

One hundred seven eyes (52 right eyes) of 93 patients (46 female patients) were included in the study (Table 1). There was no patient for whom the VERION registration system failed to calculate the degree of cyclotorsion. The mean age ± the standard deviation of the patients was 71.7 ± 10.6 years (range, 40–91 years). The mean corneal astigmatism was 1.04 ± 0.63 diopters (range, 0.06–3.00 diopters). The mean axial length (AL) was 24.41 ± 1.87 mm (range, 21.21–30.80 mm). The mean white-to-white corneal diameter (WTW) was 11.88 ± 0.53 mm (range, 9.79–13.00 mm).

**Table 1:** Characteristics of study subjects.

Characteristics	Mean ± SD
Total, eyes (patients)	107 (93)
Right / Left, eyes (%)	52 (48.6)/55 (51.4)
Female, eyes (%)	46 (43.0)
Age, years	71.7 ± 10.6
Axial length, mm	24.41 ± 1.87
WTW, mm	11.8 ± 0.53
Corneal astigmatism, diopters	1.04 ± 0.63
**Visual acuity, LogMAR**	
Preoperative UCVA	0.90 ± 0.42
Preoperative CVA	0.32 ± 0.27
Postoperative UCVA	0.34 ± 0.41
Postoperative CVA	-0.04 ± 0.14
**Visual acuity of fellow eyes, LogMAR**	
Preoperative UCVA	0.61 ± 0.50
Preoperative CVA	0.11 ± 0.27
Duration of operation, minute	9.36 ± 1.57

SD = standard deviation; WTW = white-to-white corneal diameter; LogMAR = logarithm of the minimum angle of resolution; UCVA = uncorrected visual acuity; CVA = corrected visual acuity.

### Posture-related ocular Cyclotorsion

**Figure 1:**
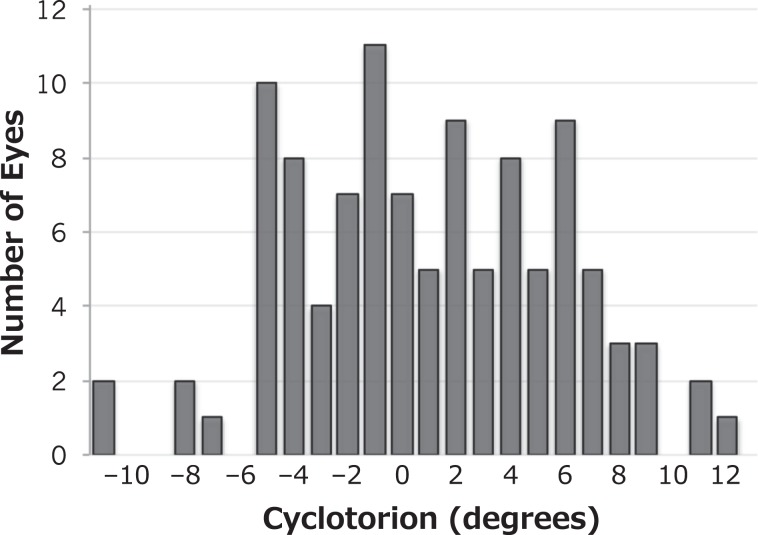
Distribution of posture-related ocular cyclotorsion A positive value and negative value of cyclotorsion indicate excyclotorsion and incyclotorsion, respectively. Posture-related cyclotorsion ranged from −11 to 12 degrees. Cyclotorsion was ≥3 degrees in 68 (63.6%) of 107 eyes. There was no cyclotorsion in seven (6.5%) eyes. Excyclotorsion occurred more frequently than incyclotorsion (50.5% vs. 43.0%).

The distribution of cyclotorsion between the seated and the supine positions in this study is presented in Fig. 1. The mean cyclotorsion was 0.98 ± 4.85 degrees (median, 1 degree; range, -11 to 12 degrees). The median absolute value of cyclotorsion was 4 degrees (mean, 4.05 ± 2.82 degrees). The cyclotorsion was 3 degrees or more in 68 (63.6%) eyes, 5 degrees or more in 43 (40.2%) eyes, and 10 degrees or more in five (4.7%) eyes. Excyclotorsion occurred in 54 (50.5%) eyes and incyclotorsion in 46 (43.0%) eyes. No cyclotorsion occurred in seven (6.5%) eyes.

In 52 right eyes, the mean cyclotorsion was 1.83 ± 4.71 degrees (median, 2 degrees; range, -7 to 12 degrees): excyclotorsion occurred in 29 (55.8%) eyes; incyclotorsion occurred in 20 (38.5%) eyes; and no cyclotorsion occurred in 3 (5.8%) eyes. In 55 left eyes, the mean cyclotorsion was 0.18 ± 4.89 degrees (median, 0 degree; range, -11 to 11 degrees), with excyclotorsion in 25 (45.5%) eyes, incyclotorsion in 26 (47.3%) eyes, and no cyclotorsion in 4 (7.3%) eyes.

### Bilateral cataract surgery

Fourteen patients (representing 28 eyes) underwent bilateral cataract surgery on separate days. Five (35.7%) patients had bilateral excyclotorsion and two (14.3%) patients had bilateral incyclotorsion. Seven (50.0%) patients had bilateral clockwise or counterclockwise rotation. The 14 patients had no significant correlations in the degree of cyclotorsion between the right and left eyes (*r = *-0.24, *P* = 0.41).

### Predictive factors for cyclotorsion

**Figure 2:**
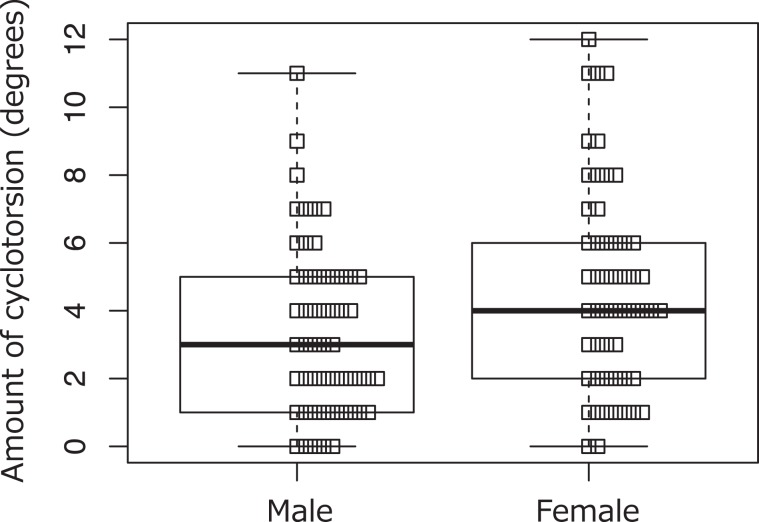
The absolute value of cyclotorsion in male and female patients The mean absolute value of cyclotorsion is 4.66 ± 3.02 degrees in female patients and 3.44 ± 2.52 degrees in male patients (median, 4 degrees and 3 degrees, respectively). Female patients have significantly larger posture-related cyclotorsion than do male patients (*P* = 0.039).

Multiple regression analysis was used to explore potential variables that influence the absolute value of posture-related cyclotorsion. Gender, AL, and preoperative corrected visual acuity (pre-CVA) were chosen as the predictive variables. A significant regression equation was found [*F* (3,103) = 2.10, *P* = 0.023] with an *R*^2^ of 0.23. The patients’ predicted absolute value of cyclotorsion was

$$6.20+1.06({\rm{GENDER}})-0.16({\rm{AL}})+0.36({\rm{PRE}}-{\rm{CVA}})$$

in which gender is coded as 1 = male and 2 = female. The standardized partial regression coefficient (1.06) was significant only for gender [*t* (103) = 1.90; *P* = 0.041]. The mean absolute value of cyclotorsion was significantly larger in female patients than in male patients [median, 4 degrees and 3 degrees, respectively; mean, 4.66 ± 3.02 degrees and 3.44 ± 2.52 degrees (*P* = 0.039)] (Fig. 2).

## Discussion

In this study, we measured posture-related cyclotorsion in phacoemulsification surgery using an ocular registration system and found that 68 (63.6%) eyes had cyclotorsion of 3 degrees or more. With regard to the correction of astigmatism in cataract surgery, posture-related cyclotorsion was clinically significant, especially in toric IOL implantation.

Nearly all previous studies on posture-related cyclotorsion only targeted normal young individuals or patients undergoing corneal refractive surgery. We believe that posture-related cyclotorsion in cataract surgery and corneal refractive surgery may have different features because the patients had different demographics. For example, the mean age of patients undergoing cataract surgery was approximately 70 years, whereas patients undergoing LASIK and PRK were younger than 30 years (Table 2). As a result, cyclotorsion of 5 degrees or more occurred in 43 (40.2%) eyes undergoing cataract surgery, whereas cyclotorsion occurred in approximately 10–30% of eyes undergoing corneal refractive surgery^5,15–18,21^ (Table 2). This finding suggested that cataract patients may experience large-angle cyclotorsion more frequently than patients undergoing LASIK or PRK.

**Table 2:** Studies of posture-related ocular cyclotorsion using an ocular registration system.

Study	Eyes	Mean age (year)	Mean absolute cyclotorsion (degrees)	Eyes with cyclotorsion ≥5 degrees (%)	Eyes with excyclotorsion (%)	Surgical procedure
Kim *et al*. ^21^	140	27.2 ± 5.6	2.59 ± 1.91	13.0	54.3	LASIK
Febbraro *et al*. ^16^	74	29.6	3.08 ± 2.68	29	60	LASIK
Liu *et al*. ^15^	186	29.1	3.22 ± 2.53	21.0	60.8	LASIK or PRK
Hummel *et al*. ^24^	337	68.0 ± 9.0	5.81 ± 4.20	39.5 (≥6 degrees)	30.9	FLACS
Present study	107	71.7 ± 10.6	4.05 ± 2.82	40.2	50.5	PEA with IOL

LASIK = laser *in situ* keratomileusis; PRK = photorefractive keratectomy; FLACS = femtosecond laser-assisted cataract surgery; PEA = phacoemulsification and aspiration; IOL = intraocular lens.

The direction and maximum value of cyclotorsion in our patients were comparable to those reported in previous studies. The tendency of frequent excyclotorsion in this study has been previously reported^5,15,16,18,21^. Febbraro *et al*.^16^ and other investigators^15,16,18^ reported that the maximum cyclotorsion was approximately 10–15 degrees, whereas the maximum cyclotorsion was 12 degrees in the current study.

Based on our data, it was quite difficult to predict the absolute value of cyclotorsion accurately from independent variables because of the low coefficient of determination in our regression model. However, gender significantly influenced cyclotorsion. In addition, the mean absolute value of cyclotorsion was significantly larger in female patients than in male patients. Prakash *et al*.^27^ reported that dynamic cyclotorsion during LASIK depended on gender. They showed that the absolute range of dynamic cyclotorsion was larger in female individuals than in male individuals. Dynamic cyclotorsion is small torsional movements in the supine position occurring during the treatment procedure, whereas posture-related cyclotorsion is classified as static cyclotorsion. Dynamic cyclotorsion and static cyclotorsion are different types of rotational movement; however, these two types of eye movements may have a common feature.

The mechanism of posture-related cyclotorsion remains unclear. Certain factors can cause cyclotorsion such as vestibular system function and eyeball reposition with orbital tissue after postural change. Furuta^28^ and Wei *et al*.^29^ demonstrated that gender differences in the orbital volume is significant after the age of 12 years, and male individuals have significantly larger orbital volumes than do female individuals. Naylor and McBeath^30^ found that female individuals more strongly utilize auditory information for the perception of body tilt, compared to male individuals. Furthermore, Regensburg *et al*.^31^ demonstrated that the volume of extraocular muscles was larger in male than in female, and the volume remained stable in male but decreased in female with increasing age. These anatomical and functional gender differences may have lead to our findings that female patients had larger absolute values of posture-related cyclotorsion than did male patients.

In this study, excyclotorsion occurred more frequently than incyclotorsion in right eyes (55.8% and 38.5%, respectively). On the contrary, incyclotorsion occurred more frequently than excyclotorsion in left eyes (47.3% vs. 45.5%). These facts probably led to the difference of the mean cyclotorsion between right and left eyes (1.83 and 0.18 degrees, respectively), although not statistically significant. Zhao *et al*.^25^ reported that the difference of cyclotorsion might be correlated with eye laterality, and excyclotorsion was predominant in right eyes, whereas incyclotorsion was predominant in left eyes, which was consistent with our results. As the reason for the findings, they focused on a common knowledge that in most people the right eye was the dominant eye, and hypothesized that the muscles of the dominant eye might be stronger than those of the other eye, resulting in the bilateral difference.

Posture-related cyclotorsion is a main cause of cyclotorsion, but other factors can also cause cyclotorsion such as the unmasking of cyclophoria by monocular occlusion^11^ and eyeball distortion due to the lid speculum. Moreover, head tilt could have induced measurement errors in cyclotorsion^14^.

A limitation of this study was that cyclotorsion caused by certain factors—except for postural change—and the noncyclotorsional component due to head tilt may affect the direction and degree of posture-related cyclotorsion. For reducing the contamination of head tilt, we aligned the height of right and left eyes when a preoperative image was obtained in the seated position. In addition, this study could not verify the reliability of the VERION registration system, compared with that of other ocular registration systems or conventional methods. A further comparative study is needed. Finally, we believe that other predictive factors should be added as independent variables to improve the explanatory power of the regression equation. Adib-Moghaddam *et al*.^32^ showed that contrast sensitivity and visual axis indices had an effect on intraoperative ocular cyclotorsion.

In conclusion, we found significant posture-related cyclotorsion in cataract patients. Gender impacts the absolute value of cyclotorsion. Moreover, cataract patients experienced large-angle cyclotorsion more frequently than did patients who underwent LASIK and PRK.

## Methods

We retrospectively reviewed a consecutive series of eyes with a cataract, which was treated with phacoemulsification and aspiration (PEA) with IOL implantation between January and May 2016, and in which cyclotorsion was evaluated using the VERION Image Guided System (Alcon Laboratories). Two surgeons (T.O. and H.T.) performed all surgeries at the Department of Ophthalmology at the Jikei University School of Medicine (Tokyo, Japan). In accordance with the routine procedure in our hospital, all patients scheduled to undergo surgery were provided a thorough explanation of the risks and benefits of surgery, including a discussion of nonsurgical alternatives. Informed patient consent was then obtained. This study was approved by the institutional review board of the Jikei University School of Medicine (approval number: 30–142(9163)). Informed consent for the use of medical record was obtained from all patients, and patient data were used in accordance with the tenets of the Declaration of Helsinki.

### Patients’ examinations

The variables extracted from the clinical charts included age, gender, corneal astigmatism, WTW, AL, laterality of eyes, preoperative uncorrected visual acuity (pre-UCVA), preoperative corrected visual acuity (pre-CVA), duration of operation, and the degree and direction of cyclotorsion when the patient changed from the seated to supine position. The WTW was measured as the transverse diameter of the limbus. Corneal astigmatism and the WTW were measured using the VERION Reference Unit. The AL was measured using the IOLMaster 700 optical biometer (Carl Zeiss Meditec, Inc., Dublin, California, USA). The decimal visual acuity obtained using the Landolt C chart was converted to the logarithm of the minimum angle of resolution scale.

### Cyclotorsion measurements

The degree and direction of cyclotorsion were measured using the VERION Image Guided System. With the patient seated, the VERION Reference Unit captured the patient’s scleral vessels, limbus, and iris features. When the image was captured, the patient’s head was maintained in an upright position, and the height of the right and left eyes were aligned to prevent head tilt. At the beginning of cataract surgery, the patient was placed supine on the surgical table. The VERION system matched the eyes of the patient when supine to the preoperative image obtained when the patient was seated, and immediately calculated the degree and direction of cyclotorsion.

### Statistical analyses

Clinical findings were statistically evaluated using R software (version 3.6.1; The R Foundation; http://r-project.org). A positive value and negative value of cyclotorsion indicate excyclotorsion and incyclotorsion, respectively. The mean cyclotorsion indicates the average of these positive or negative values. The absolute value of cyclotorsion indicates the amount of change represented by a positive value regardless of the direction of cyclotorsion. Mann–Whitney *U* test was used to compare continuous variables between two groups. Pearson’s correlation analysis was used to assess the strength of the relationship between two variables. Multiple regression analysis was used to predict the absolute value of cyclotorsion, based on age, gender, corneal astigmatism, WTW, AL, laterality of eyes, pre-UCVA, pre-CVA, and duration of operation. A *P* value less than 0.05 was considered as statistically significant.
